# Integrated Pest Management Strategies for Asian Citrus Psyllid *Diaphorina citri* Kuwayama (Hemiptera: Psyllidae) and Huanglongbing in Citrus for Sarawak, East Malaysia, Borneo

**DOI:** 10.3390/insects13100960

**Published:** 2022-10-20

**Authors:** Sui S. Leong, Stephen C. T. Leong, George A. C. Beattie

**Affiliations:** 1 Faculty of Agricultural and Forestry Science, Universiti Putra Malaysia Bintulu Sarawak Campus, Nyabau Road, Bintulu 97008, Sarawak, Malaysia; 2 Institute of Ecosystem Science Borneo, Universiti Putra Malaysia Bintulu Sarawak Campus, Nyabau Road, Bintulu 97008, Sarawak, Malaysia; 3 School of Science, Western Sydney University, Locked Bag 1797, Penrith, NSW 2751, Australia

**Keywords:** citrus, *Diaphorina citri*, ‘*Candidatus* Liberibacter asiaticus’, huanglongbing, insecticides, integrated pest management

## Abstract

**Simple Summary:**

The severe Asian form of huanglongbing is the most serious disease of citrus in Malaysia. It is caused by a phloem-limited bacterium ‘*Candidatus* Liberibacter asiaticus’ and transmitted by the Asian citrus psyllid, *Diaphorina citri* Kuwayama. Minimising the devastating impacts of the disease requires effective integrated pest management (IPM) programs to limit detrimental environmental and health consequences of over-reliance on synthetic pesticides. Key components of such programs include grower awareness, planting of pathogen-free trees, monitoring psyllid and disease incidence, removal of diseased trees, and judicious use of insecticides, mineral oils, and natural enemies to suppress incidence of the psyllid. Significant progress has been made towards implementing the programs.

**Abstract:**

The Asian citrus psyllid (ACP), *Diaphorina citri* Kuwayama, transmits ‘*Candidatus* Liberibacter asiaticus’ (*C*Las), a phloem-limited bacterium associated with the severe Asian form of huanglongbing (HLB), and the most destructive disease of citrus. The pathogen and the psyllid, both of South Asian origin, are now widespread in citrus regions of Asia and the Americas. There is no cure for the disease. Application of synthetic pesticides, in some instances more frequently than fortnightly, to minimise incidence of ACP in citrus orchards, has not prevented inevitable impacts of the disease in regions of Asia where *C*Las is present. Despite the inevitable spread of the disease, significant progress has been made in Sarawak since the mid-1990s towards effectively implementing integrated pest management (IPM) programs for stemming the impact of the disease and detrimental consequences of over-reliance on synthetic pesticides. Growers are encouraged to plant pathogen-free trees, remove diseased trees, monitor incidence of the psyllid, and to use pesticides judiciously to reduce their detrimental impacts on natural enemies. Knowledge has been enhanced through research on seasonal incidence of the psyllid, use of mineral oils, development of protocols and iodine–starch test kits for detecting infected trees, PCR for confirming the presence of *C*Las in symptomatic leaves, methods for monitoring incidence the psyllid, and training extension staff and growers. However, major impediments to increasing the average longevity of trees beyond <5 years in poorly managed orchards, based on marcotting (air layering), and >12 years in well-managed orchards, based on pathogen-free trees, still need to be addressed. These include grower knowledge, marcotting, aggressive marketing of synthetic pesticides, high prices of mineral oils, spray application procedures, and better reliance on natural enemies of the psyllid.

## 1. Introduction

The genus *Citrus* (Rutaceae: Aurantioideae: Aurantieae) includes species and hybrids of widely cultivated fruits (grapefruit, kumquats, lemons, limes, oranges, and pomelos) in tropical and subtropical regions of the world [1,2,3,4]. The genus is believed to have originated from the region comprising Northeast South Asia, South and Southwest China, Indochina, and Malesia [5]. In Malaysia, citrus is grown in commercial orchards, small holdings, and backyards. It is an important fruit crop in the East Malaysian state of Sarawak in northern Borneo, where it provides a livelihood to many growers who supply mainly to the domestic market. The total area of commercial orchards in the state was approximately 3250 ha in 2005 [6]. An estimated 28.35 metric tonnes of fresh orange fruit/juice were exported to Singapore, Peninsular Malaysia, and Brunei in 2005. The most important production areas in Sarawak are located in the Samarahan Division adjacent to the state capital Kuching (1.5548° N, 110.3594° E), with some major plantings in Sarikei, Sibu, and Bintulu Divisions, some 140, 190, and 350 km, respectively, northeast of Kuching.

The major impediments to production are the severe Asian form of huanglongbing (HLB) putatively associated with a phloem-limited Gram-negative bacterium ‘*Candidatus* Liberibacter asiaticus’ (*C*Las) (α-Proteobacteria) and the Asian citrus psyllid (ACP), *Diaphorina citri* Kuwayama (Hemiptera: Sternorrhyncha: Psyllidae), the most widely distributed vector of the pathogen that has severely affected citrus production in Asia and the Americas [7,8,9,10,11,12]. The psyllid was noted as being present in Malaysia by Clausen [13]. Symptoms resembling HLB were first noticed in Malaysia in the 1970s in lowland peninsular orchards near Kampung Jerangau (4.8370° N, 103.1971° E, 20 m above sea level (ASL); Kuala Dungun, in Terengganu; Ulu Tiram, in Johor (1.5974° N, 103.8150° E, 25 m ASL); and the highland area near Ringlet (4.4146° N, 101.3846° E, 1110 m ASL) in the Cameron Highlands in Pahang [14,15,16,17], but no bacterium-like organisms were observed in sectioned fixed leaves examined under an electron microscope in France [18]. Broadbent [18] observed symptoms typical of the disease in grapefruit (*Citrus ×* *aurantium* L.), sweet orange (*C. ×* *aurantium*), and mandarin (*C. ×* *aurantium*) leaves in the Cameron Highlands. Grapefruit were severely affected while lemons (*C. ×* *limon* (L.) Osbeck), limes (Mexican, Key, alemow: *C. ×* *aurantiifolia* (Christm.) Swingle, syn. *C. ×* *macrophylla* Wester), and Dancy tangerine (*C. ×* *aurantium*) appeared to have some tolerance. However, no bacterium-like organisms were observed in sections of young leaves [18]. Symptoms were also reported from the Cameron Highlands by Catling [19]. Information provided by Ko [20] and Saamin et al. [21] suggests that the first record of the disease in Peninsular Malaysia was related to the introduction of *C. × aurantium* seedlings, in this instance Tanaka’s ‘*C. suhuiensis*’, a mandarin variety from Sihui (23.3534° S, 112.8964° E ), near Guangzhou in Guangdong, mainland China, to Terengganu, from China in the 1950s and 1960s. The presence of the disease was confirmed when the pathogen *C*Las was detected in sectioned leaves from symptomatic plants in the Cameron Highlands in 1987 [22,23,24]. The disease was first detected in Sarawak in the Samarahan Division in 1988 [25]. Since then, research and extension activities undertaken by the Sarawak Department of Agriculture (SDOA) and universities in the state have focused on minimising the impact of the disease. Intensive pesticide programs lead to insecticide resistance and are harmful to pollinators and beneficial insects. Therefore, it is important to design IPM strategies that minimise the utilisation of pesticides, especially neonicotinoids and other broad-spectrum materials, and maximise the use of natural enemies, especially the primary ectoparasitoid *Tamarixia radiata* (Waterston) (Hymenoptera: Eulophidae) and the primary endoparasitoid *Diaphorencyrtus aligarhensis* (Shafee, Alam and Argarwal) (Hymenoptera: Encyrtidae), as well as several predators, including the weaver ant, *Oecophylla smaragadina* Fabricius (Hymenoptera: Formicidae). We review literature pertinent to ACP and *C*Las in Malaysia and discuss progress, goals, and constraints for implementing integrated pest management (IPM) programs for the psyllid and HLB in Sarawak while maintaining ecological, environmental, and economic sustainability.

## 2. Origin and Distribution of Asian HLB

Although the disease was first recognised as transmissible in China [8], the first unambiguous description of symptoms is within text in which Husain and Nath [26] describe damage caused by the psyllid: small and insipid fruit, dieback, and death of trees caused by a ‘toxin’ injected into plant tissues by the psyllid. The pathogen probably originated in association with a non-citrus host in South Asia. It did not originate in mainland China or Taiwan. The disease was not described, as oft cited, by Reinking [27], who observed citrus maladies in Guangdong in mainland China, or in Taiwan by Sawada (1913) [28], as claimed recently by Zhou [29] and Guo et al. [30]. The first authentic record of HLB in mainland China appears to have been made in Guangdong in 1938 [31,32], four years after ACP was recorded in the province [33,34,35]. HLB was first recorded in Taiwan in 1951 [35,36]. It is now widespread in Asia, the Americas, and is present in the Mascarene Islands, New Guinea, and, most recently, Africa [7,37,38,39,40,41,42].

### Host Susceptibility to HLB

All citrus species and hybrids are susceptible to the Asian form of HLB, although some species, e.g., *C. cavaleriei* H. Lév. ex. Cavalerie (China), *C. gracilis* Mabb. (Australia), and *C. hystrix* DC (Indochina and Malesia), have not been evaluated or fully evaluated. The least susceptible species, in approximate order of increasing susceptibility, with their geographic origin in parentheses, include *C. glauca* (Lindl.) Burkill (Australia); *C. halimii* B.C. Stone (Malaysia and Thailand); *C. australasica* F. Muell., *C. inodora* F. M. Bailey, and *C. × virgata* Mabb. (Australia); *C. (Poncirus) trifoliata* L. (China); *C. × oliveri* Mabb. (*C. australasica* × *C. × microcarpa* Bunge) and *C. australis* (A.Cunn ex Mudie) Planchon (Australia); and *C. latipes* (Swingle) Tanaka (Northeast South Asia) [43,44]. The susceptibility to CLas of species of *Citrus* (*Oxanthera*) from New Caledonia and of *C. polyandra* Tan. (syn. *Clymenia polyandra* (Tan.) Swingle) from New Ireland in Papua New Guinea has not been determined.

In Malaysia, Shokrollah et al. [45] assessed the susceptibility of 18 citrus ‘species’ to HLB following graft transmission of *C*Las from infected mandarin plants. The pathogen was detected by PCR in 15 of the species 6 months after grafting. Susceptibility was categorised into five symptom groups: severe (72–58% severity), comprising ‘limau madu’ and Cleopatra mandarins, and sweet orange; moderate (50–41% severity), comprising kumquat (*C.* (*Fortunella*) *japonica* Thunb. cv. Kasturi Chinai), *C. aurantiifolia* (cv. ‘machrophylla’), and calamondin (*C. × microcarpa* Bunge); mild (25–17% severity), comprising citron (*C. medica* L.), *C. aurantiifolia*, *Citrus* sp. (natural genotype), and rough lemon (*C. ×* *otaitensis* (Risso and Poit.) Risso syn. *C. jambhiri* Lush.); tolerant (no symptoms but PCR positive), comprising sour orange (*C. × aurantium*) and *C. aurantiifolia* (cv. ‘limau nipis’)); and resistant (no symptoms and PCR negative), comprising pomelo (*C. maxima* (Burm.) Merr., cv. limau Bali), limau purut or leech lime (*C. hystrix*), and *Citrus* sp. (cv. ‘Limau Tembikai’).

*C*Las-infected trees become unproductive (30–100% reduction in yield) and may die in 5–8 years, or less, of infection, depending on the susceptibility to the disease of the host plant species/hybrid [8]. Short lifespans (<3 years) of trees are often related to marcotting (air layering) (Beattie, personal observation). There is currently no cure for HLB, the best control strategy being the prevention of the disease [8]. Movement of infected citrus material by land, sea, and air is the most efficient way of disseminating HLB over large distances. Entry of *C*Las to parts of Southeast Asia and Malesia, and to the Mascarene Islands, has been linked to human migration and movement of plants from China, after the pathogen was introduced to China [10,39,46,47]. The use of disease-free planting material from reliable sources is the first and most important step towards good disease management. Mother trees from which budwood is obtained must be kept in well-maintained insect-proof screenhouses.

Initial lack of awareness of this disease, slow introduction of diagnostic tests, and rapid propagation of non-certified planting material by citrus growers to expand their orchards have contributed to poor management of HLB in Malaysia. The reluctance of growers to remove infected trees due to limited knowledge about the pathogen, its mode of spread by ACP, and the cost of vector control, have contributed to the problem. Surveys in 2004 by Azizah and Zazali [48] revealed that approximately 70% of the cultivated area (3526 ha) of citrus in Peninsular Malaysia was affected. In Terengganu, the area affected increased from 641 ha in 2001 to 1262 ha in 2004 [48].

HLB in Sarawak destroyed 1143 ha of orchards, comprising ‘limau langkat’ (*C. × aurantium*, Tanaka’s ‘*C. suhuiensis*’) or mandarin orange, sour or bitter orange, and leech lime, in the Samarahan Division by 1991. Economic losses in the state were estimated to be 6.5 million Malaysian ringgit (RM) (1.6 million United States dollar (USD) [49,50,51]. Citrus fruits had to be imported from neighbouring countries, such as Indonesia and Thailand, to meet the local demand. After the decline of the citrus industry in the Samarahan Division, the SDOA started producing disease-free planting material in 1996. The disease-free planting material was produced using budwood obtained through shoot tip micro-propagation [52].

## 3. Origins and Distribution of ACP

ACP originated on the Indian subcontinent. Spread of the psyllid eastward to Southeast and East Asia appears to have been linked to movement of infested host plants from Southern India and Sri Lanka to Java in Western Indonesia, and subsequently to Ambon and Timor in Eastern Indonesia, Luzon in the Philippines, Macao, and Taiwan [46]. Although ACP was first described from specimens collected from citrus in ‘Shinchiku Prefecture’ in northern Taiwan [53], then Japanese occupied ‘Formosa’, it was first recorded in Java in 1900 [46]. The psyllid now occurs in South Asia, East Asia, Malesia, New Guinea in Australasia, Arabia and Israel in West Asia, Caribbean islands; North, Central and South America; some Pacific Ocean islands; and the Maldives and Mascarene islands in the Indian Ocean [46]. It was recorded in Africa in 2015 [54] and was recently recorded in Israel in 2021 [55].

### 3.1. Host Susceptibility to ACP

Known hosts of ACP were reviewed by Beattie [56]. Among 87 seed-source field-planted seedlings of 76 *Citrus* species and hybrids, and 11 *Citrus* relatives colonised by ACP adults over 4 months in summer and early autumn in Florida, the least susceptible *Citrus* species and hybrids were *C. trifoliata*, *C. inodora*, *C. glauca*, *C. halimii*, *C. australasica*, *C. × virgata*, *C. × leiocarpa* hort. ex Tan., *incertae sedis* (Koji mandarin), and sour orange. The most colonised, including *Citrus* relatives, were *C. reticulata*, *Bergera koenigii* L. (curry leaf) (Aurantioideae: Clauseneae), *Murraya paniculata* (L.) Jack (orange jasmine) (Aurantieae), *C. medica*, and *C. aurantiifolia* (lime, Key or Mexican lime, alemow) [57]. Lim et al. (1990-citrus greening) [24] cited *C. hystrix*, *C. maxima*, *C. reticulata* L. (including *C. suhuiensis*), *C. × aurantiifolia*, *C. × limon*, *C. × microcarpa* Bunge, Rangpur lime (*C. ×* *otaitensis*), *B. koenigii* and *Clausena excavata* Burm.f. (Clauseneae), and *M. paniculata* as hosts in Malaysia. In Sarawak, Leong et al. [57] reported that ACP can colonise and breed on ‘limau langkat’, orange jasmine, and curry leaf, and that orange jasmine is the preferred host. ACP completes its life cycle (from egg to adult) in the shortest period (18.5 days) on orange jasmine, followed by ‘limau lankat’ (19.0 days) and curry leaf (23.0 days) [57]. The susceptibility of species of *Citrus* (*Oxanthera*) from New Caledonia and *C. polyandra* from Papua New Guinea as hosts of ACP has not been determined.

### 3.2. Biology of ACP and Influence of Abiotic Factors

ACP has a high capacity to increase in numbers on highly suitable hosts in monocultures [26,58,59], but in the absence of HLB it is a minor pest. It is not able to build up massively on rutaceous host trees or shrubs in forests [59] but is capable of surviving in low-density populations in natural habitats on sparsely spaced host plants with limited immature flush growth suitable for oviposition and development of nymphs [59]. Liu and Tsai [60] studied development, survival, longevity, reproduction, and life table parameters of ACP in growth chambers at temperatures ranging from 10 °C to 33 °C and relative humidities between 75 and 80%. Populations reared at 10 °C and 33 °C failed to develop [60]. However, populations can flourish in Köppen–Geiger BWh desert and BSh hot semi-arid zones of South Asia, where average daily maximum temperatures over several weeks can reach 45–50 °C [46]. The effects of different temperatures on the life history parameters, including development times and longevity for different geographic populations, have been assessed several times [60,61,62,63,64,65]. A meta-analysis of published ACP temperature-dependent development literature by Milosavljevic et al. [65], synthesising datasets of five globally distributed populations (Brazil, California, China, Florida, and Japan) reared under different constant temperatures on six different host plants (i.e., Rangpur lime, Natal and Pêra oranges, kumquat, mandarin, and orange jasmine), together with the results of the study on Volkamer lemon (*C. × otaitensis*), revealed convergence in estimates of developmental parameters. These results have implications for predicting ACP invasions and establishment risks, and subsequent population dynamics across various climactic gradients and geographic regions. Ahmad [66] estimated that >4.5 million adults and nymphs were present in March (spring) 1959 in a one-acre (0.4 ha) irrigated orchard comprising 110 mature *C. × aurantiifolia* trees near Multan (30.1981° N, 71.4685° E, 129 m ASL) in the hot-desert Punjab region of Pakistan. Husain and Nath [26] recorded up to 807 eggs per female, but in other studies, fecundity ranged from 180 to near 1900 eggs per female, depending on the ambient conditions of the host plants [60,67,68,69]. Heavy rainfall, particularly monsoon rains, washes eggs and nymphs of plants [46]. Aubert [9,10,11] mentioned that ACP mortality increases with higher rainfall and relative humidity, and that monthly rainfall above 150 mm is associated with low populations due to eggs and young nymphs being washed off plant surfaces.

ACP has been recorded at elevations up to 1250 m above sea level near Kuala Terla (4.5467° N, 101.4162° E) in the Cameron Highlands in Peninsular Malaysia [23,24]. Massive populations were observed on HLB-infected trees in a neglected orchard, but no psyllids were observed on infected trees at 1450 m ASL [23,24]. Om et al. [70] rarely recorded it at elevations above 1200 m in Bhutan and suggested that UV-B, which increases with increasing elevations above sea level, may affect survival of the psyllid. They found no relationship between abundance of the psyllid at elevations ranging from 800 to 1500 m and tree growth, ambient air temperatures, relative humidity, rainfall, or natural enemies [70,71].

## 4. Natural Enemies of ACP in Asia

Natural enemies of ACP in Asia include the primary parasitoids *T. radiata* and *D. aligarhensis*, predators such as ladybirds (Coleoptera: Coccinellidae), lacewings (Neuroptera: Chrysopidae), ants (Hymenoptera: Formicidae), thrips (Thysanoptera: Phlaeothripidae), syrphid flies (Diptera: Syrphidae), bugs (Hemiptera: Geocoridae), mantids (Mantodea: Mantidae), mites (Acari: Phytoseiidae), and spiders (Acari: Clubionidae, Gnaphosidae and Salticidae), and six species of entomopathogenic fungi [46]. The importance of *O. smaragadina* has been overlooked [72]. It occurs naturally in Borneo and elsewhere in tropical and subtropical citrus regions in Asia, New Guinea, and northern Australia, and has been recognised as an important predator of citrus pests in Asia, particularly China and Vietnam, for some 1700 years [71]. It preys on eggs of ACP, and incidence of psyllid and HLB has been recorded as lower in unsprayed orchards than in orchards sprayed with synthetic pesticides [72,73].

## 5. Transmission of ‘*Candidatus* Liberibacter asiaticus’ (CLas) by ACP

Both nymphs (fourth and fifth instars) and adults are able to acquire and transmit *C*Las, but adults, because they fly, are significant contributors to the spread of HLB [74,75,76,77]. ACP is an efficient insect vector of HLB’s causal pathogen, *C*Las, in Asia and the Americas, and high ACP populations in HLB-resident areas lead to a high incidence and spread of HLB. DNA hybridisation disclosed that the percentage of viruliferous ACP adults observed in the affected citrus grove ranged from 5% to 39%, respectively, during May and September in Sarawak, Malaysia [78]. The establishment of ACP in a region makes its complete eradication not feasible or even desirable (highly unlikely). With one exception, the fortuitous eradication of ACP in the Northern Territory of Australia during a successful campaign in the early 1900s to eradicate citrus canker (*Xanthomonas citri* subsp. *citri* (*ex* Hasse 1915) Gabriel et al. 1989 (Pseudomonadales: Pseudomonadaceae)) [79], eradication of the psyllid once it is established in a region has not been achieved. The only option after the psyllid becomes established in a region is to keep populations in orchards as low as possible to minimise the spread of HLB. In the field, build-up of ACP populations depends on prevailing ambient temperatures and new flush. Female adults must feed on new flush to mature eggs and lay eggs on tender flush shoots and new sprouts, which ensures a continuous supply of appropriate habitat for nymph development in subsequent days [80,81,82,83,84,85]. The production of new flush, on the other hand, depends on tree age, weather conditions, and citrus variety [79]. Leaf age also influences feeding behaviour, and tissues in which the psyllid feeds [86,87]. Psyllids can be tested to assess the prevalence of *C*Las in a population and for early detection of HLB in new areas being invaded by the psyllid. There is a need for rapid and inexpensive field detection methodology.

## 6. Hosts of ACP in Sarawak

More than 16 citrus species or hybrids are grown in Sarawak. They include honey mandarin, limau langkat, king orange, tambun pomelo, sweet orange cultivars, neck orange,), sunki (wild or sour mandarin, perhaps *C. reticulata* Blanco var. *austera* Swingle, and thus *C. reticulata*, *sensu stricto* [1]), lime (*C. aurantiifolia*), calamondin, leech lime, lemons, Rangpur lime, Cleopatra mandarin (‘*C. reshni* Tan.’), timkat mandarin (‘*C*. *oleocarpa* Tan.’), and citrumelo and citrange trifoliate hybrids (*C. × insitorum* Mabb.).

Orange jasmine and curry leaf are widely grown alternative hosts, and if found in proximity to a citrus orchard they should be removed as part of the cultural practice for ACP control. *Murraya sumatrana* Roxb., recently recognised as one of four species currently comprising the *Murraya paniculata* Complex [87], also occurs naturally on limestone in Sarawak and elsewhere in Borneo, but its status as a host of ACP is not known, possibly due to it being widely regarded until recently as *M. paniculata* [88].

## 7. Citrus IPM in Sarawak: Goals and Constraints

Successful IPM strategies for ACP and HLB in Sarawak require propagation, distribution, and planting of pathogen-free trees, detection and monitoring of psyllid populations and disease incidence in orchards, informed growers with adequate resources, action thresholds for maintaining psyllid populations below levels of concern, effective and environmentally acceptable chemicals for suppressing psyllid populations, and effective application of insecticides, supplemented with key natural enemies of ACP and removal of infected trees when symptoms of the disease are evident in <30% of trees, and entire orchards when more than 30% of trees have symptoms. Each of these prerequisites for successful strategies is beset with constraints.

### 7.1. Disease-Free Planting Material

To rebuild the once productive citrus industry in Samarahan Division, the Sarawak Department of Agriculture (SDOA) embarked on a rehabilitation program in 1996 that adopted a general recommendation, as stated above, which is still recognised and practiced throughout the world, for successful management of HLB. Several senior SDOA officers were trained in Taiwan and France on the production, management, and certification of pathogen-free material in 1994–1995. The rehabilitation program commenced in 1996, under the Seventh Malaysia Plan, with the establishment of foundation stock at the Agriculture Research Centre (ARC) at Semongok (1.1442° N, 110.3257° E) near Kuching. However, production of pathogen-free grafted plants was not successful until production was transferred to a private nursery in 1998. The SDOA continued to supply the HBL-free planting materials and insecticides to participants in its crop improvement program. Nevertheless, most private nurseries and suppliers of citrus plants, growers, and entrepreneurs of citrus orchards were still not fully aware about the danger of HLB, despite the SDOA alerting nurserymen and growers about the seriousness of the disease to the industry. This lack of awareness led to ongoing production and use of uncertified planting materials, reluctance by growers to remove diseased trees, and an outbreak of HLB in the Samarahan Division in 2001–2002. Decisions to plant marcots are often related to the cost of pathogen-free trees and ineffective means of suppressing populations of ACP below levels required to minimise spread of ‘CLas’.

**Figure 1:**
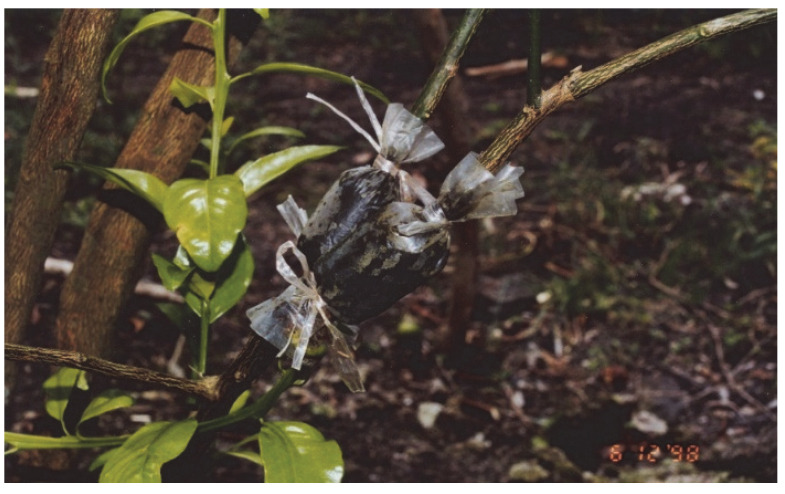
Marcotted seedlings from HLB-infected tree.

The SDOA continues to recommend the use of pathogen-free planting material from reliable sources for establishing healthy orchards or replacing trees within orchards. HLB-free seedlings propagated in Sarawak DOA insect-proof screenhouses are provided free to farmers under a scheme to rehabilitate existing citrus areas. Growers may also purchase seedlings at subsidised prices ranging from RM 14–15 (USD 3.50–3.70) from commercial nurseries for planting in existing orchards or in new planting areas. However, many growers plant *C*Las-infected marcotted seedlings produced by themselves or by other growers (Figure 1). This enhances the rapid spread of HLB between and within orchards. Levels of infection in such seedlings is high (>50%) and plants are often infested by ACP. Trees begin to die within 2 years of the symptoms of HLB appearing in the leaves. Most trees die within 5 years, bearing fruit for only 1–2 years [83]. 

Mother trees obtained from HLB-free budwood nurseries are currently kept in well-maintained, insect-proof screenhouses, located at SDOA stations at Sungai Paoh (2.0064° N, 111.4610° E) near Sarikei, Kabuloh (4.1187° N, 113.9714° E) near Miri, and ARC Semongok. HLB-free mother trees, produced by shoot tip culture from pathogen-free scion plantlets in test tubes under aseptic conditions in the laboratory, are grafted onto pathogen-free, *Phytophthora*-resistant rootstocks grown aseptically in screenhouses from seeds, to produce HLB-free mother trees. The pathogen-free planting material is produced using budwood obtained through shoot tip micro-propagation [52] and distributed to several commercial nurseries. Nyan Mei Nursery (1.4333° N, 110.3385° E), a well-established private nursery located about 2.5 km from ARC Semongok, maintains HLB-free mother trees and produces HLB-free citrus seedlings. The commercial nurseries, accredited since 2004, are monitored by the Sarawak DOA. The pathogen status of mother trees, rootstocks, and seedlings have been regularly checked by PCR at the Plant Pathology Laboratory at ARC Semongok since it was established in 2004, and the source of planting material is to be tested twice a year using PCR.

Apart from the advocacy for the use of HLB-free planting material, vector control, and the creation of public and grower awareness through workshops and seminars, rehabilitation efforts include the relocation of focal points to areas that have a lower or no infection of HLB, and where the growers are entrepreneurs. It usually takes 2–3 years for trees to bear fruit.

### 7.2. Removal of Symptomatic Trees

Growers are advised to destroy HLB-symptomatic trees by cutting their trunks near ground level and poisoning the stumps with glyphosate as soon as foliar symptoms of the disease are observed, and to destroy all trees in an orchard when symptoms of the disease are present on more than 30% of trees. This cultural practice was carried out to reduce *C*Las inoculum sources. However, in some cases growers are reluctant to remove infected trees, as they still want to harvest fruit from them even though failure to remove trees increases the rate of spread of the disease. These are difficult decisions for growers to make if they only grow citrus. Such decisions would be easier to make for growers able to plant trees in rotation with other crops. For example, when yields and fruit quality start to decline rapidly in the older block, the trees could be removed and replaced by rice as a non-host for psyllid, and new trees could be planted where rice was grown. However, the success of such a strategy would depend on the size of a farm and would be compromised if plantings are not based on pathogen-free seedlings and if incidence of *D. citri* is not suppressed. To prevent adults dispersing from symptomatic to non-symptomatic trees, growers are advised to spray infected trees with insecticides before they are removed.

Some farmers remove symptomatic branches instead of trees. This practice is discouraged, as the pruned trees are still infected and serve as reservoirs for the transmission of ‘*C*Las’ by ACP to healthy trees. Studies overseas have shown that the spread of the HLB pathogen to roots of trees from leaves on which infected adult psyllids have fed can occur before symptoms are expressed in leaves [89,90].

### 7.3. Suppressing ACP by Interplanting Citrus with Guava

Field studies in Vietnam [91,92] and Indonesia [92] in Southeast Asia have shown that infestations of ACP, and consequently incidence of HLB, were reported to be suppressed when citrus was interplanted with guava, *Psidium guajava* L. (Myrtales: Myrtaceae) [91,92,93]. These observations were made in high-density plantings of citrus that were intercropped with guava. Under such circumstances, guava volatiles may interfere with ACP’s ability to locate and infest citrus, and thus reduce feeding and oviposition by ACP on adjacent citrus trees [94,95,96,97,98]. However, impacts of interplanting appear to be limited to <2 years [92,93]. The results obtained from trials in Vietnam and Indonesia on interplanting with guava are impressive, and similar on-farm trials are to be conducted to assess its impact on ACP population and HLB. 

### 7.4. Use of Antibiotics to Reduce ‘Candidatus Liberibacter asiaticus’ Titres in Plants

The use of antibiotics for suppressing the severity of HLB and its symptoms is receiving renewed interest in Sarawak.

The commonly used antibiotic, such as penicillin or tetracycline, is applied at regular intervals (annual basis) for the continuous suppression of the HLB pathogen. However, repeated injections of tetracycline led to phytoxicity in injected citrus [99,100]. Preliminary research the on use of antibiotics (achromycin and oxytetracycline), as used in Taiwan to rehabilitate *C*Las-infected trees [101], was undertaken in Sarawak with 8–12-year-old honey mandarin trees by Eng [48]. Studies are continuing, but the use of antibiotic is not commonly practised by growers. In the studies reported by Eng [48], achromycin was found to be too phytotoxic, its use resulting in leaf fall, shoot dieback and, in certain cases, even the death of the treated trees. Oxytetracycline was less toxic. PCR tests conducted on surviving trees treated with both antibiotics showed a mixture of positive and negative results. Current research is focused on balancing optimal concentrations of the antibiotics for killing *C*Las without causing detrimental phytotoxicity. Future research may focus on several new antibiotic compounds evaluated, such as penicillin G sodium salt, oxytetracycline, and a combination product, penicillin G potassium salt + streptomycin (P + S), that have been reported to be highly effective in suppressing or eliminating *C*Las in test plant systems, with the suppression of HLB lasting for 3–14 months [96,97,102,103,104]. However, use of the antibiotics is unlikely to be economically feasible for marcotted (air layering) trees. Potential resistance to them may limit their use, and their use may require government approval due to possible negative impacts on human health (see Haynes et al.) [105].

### 7.5. Technology Transfer

Grower awareness of HLB is imperative for maintaining productive orchards. Most farmers in Sarawak are illiterate or lack the knowledge required to effectively manage citrus orchards. Education programs for nurserymen and growers include in situ training, short courses, study tours, on-farm demonstration trials, video presentations, television programs, and leaflets, brochures, and booklets. Agricultural extension staff play an important role in informing nurserymen and growers of SDOA research and development programs, and current technologies and knowledge. The emphasis of awareness programs is on encouraging the growers to plant pathogen-free seedlings, teaching them how to recognise and monitor ACP, how to effectively suppress populations of the psyllid, how to recognise visual symptoms of HLB, and when to remove infected trees.

### 7.6. Monitoring Incidence of Huanglongbing and Diaphorina citri

#### 7.6.1. Huanglongbing

Growers are encouraged to check all trees in their orchards at least seven times annually—four times in the wet season and three times in the dry season—for foliar symptoms of HLB. Extension literature is available to help them to distinguish symptoms from those caused by other maladies, including nutrient deficiencies. It is recommended that inspections should focus on mature leaves on branches in all sectors of trees, particularly branches on which leaves exhibit characteristic mottling with or without vein corking.

The iodine–starch test (IST) [49,106,107] is recommended for testing for starch accumulation in symptomatic leaves. Information regarding the test has been distributed to growers and nurseries. The technique is cheap and rapid, without a need for sophisticated equipment. A field test kit [49] has been distributed to field extension officers and major citrus growers. Tests based on three symptomatic mandarin leaves achieved 92% accuracy compared to PCR detection of *C*Las in leaf tissues. Five symptomatic pomelo leaves with vein corking are required for similar levels of accuracy [48]. Growers can send leaves to the SDOA for confirmation of test results by PCR. 

#### 7.6.2. *Diaphorina citri*

Regular monitoring of ACP populations and symptoms of HLB is essential for successful management of the psyllid and the disease. The authors estimate that <10% of growers with orchards less than 2 ha check their citrus trees frequently; generally, those with orchards larger than 2 ha monitor their orchards. The methods described below are based on studies by Eng [48] and Leong et al. [108]. Trapping and visual inspections of trees are recommended for ACP and visual inspections. Use of the IST and, where feasible, PCR, are recommended for HLB. 

**Figure 2:**
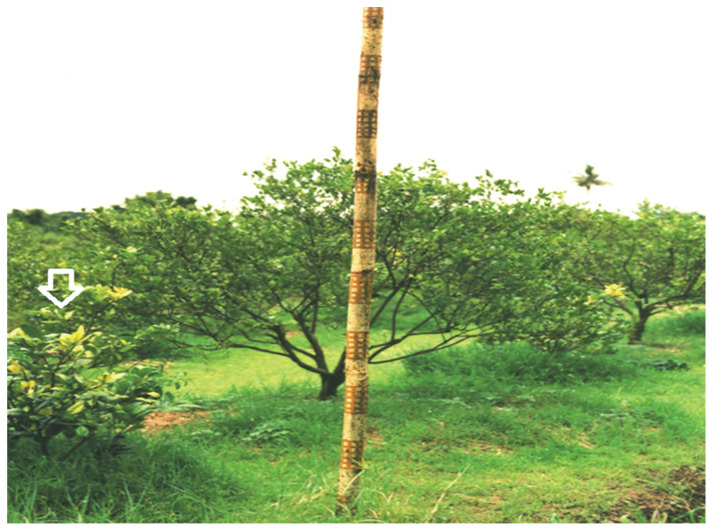
A bamboo pole trap used in the trapping of adult Asian citrus psyllid in an HLB- infected citrus orchard; an HLB-infected citrus tree showing yellow shoot symptom (arrow).

Monitoring for ACP is recommended throughout the year, but is most important in the dry season, from April to December, and should commence as soon as new flush growth appears on trees, as ambient temperatures begin to increase in April. Yellow sticky traps, brown-yellow (Rebell) sticky traps, or bamboo-pole traps [108] (Figure 2) are recommended by the SDOA for determining the presence and relative seasonal abundance of adult ACP within orchards, and the movement of adults into orchards from other farms. The traps are more attractive when there is no young leaf flush, especially after a flush has hardened, and are more effective in the dry season than in the wet season. We recommend four traps per hectare as the number required to effectively monitor ACP flight activity range from 30 m in orchards <2 ha, to 150 m in larger orchards. 

Farmer field schools can provide information about IPM and use environmentally acceptable pesticides. Growers are encouraged to visually inspect trees in their orchards regularly, particularly when trees produce new growth, which generally occurs seasonally on mature trees in February–March and September–October, and from December–March, and more frequently on immature trees. Visual inspection of new shoots for ACP eggs and honeydew produced by nymphs and adults is recommended, from bud burst to when the youngest leaves are 3–10 mm long [57]. Based on Leong et al. [109], the minimum number of flush shoots required per tree to accurately estimate ACP densities in orchards with 200 trees older than 3 years is six for eggs, four for nymphs, and two for adults. For orchards with 300 or more trees older than 5 years, a sampling plan based on randomly inspecting six flush shoots on each of 10 trees, including perimeter trees, is recommended for providing acceptable density estimates of the three life stages of ACP. However, most growers do not monitor their trees methodically and their decisions to spray are often based on ACP-life stages being observed on one to several more shoots, or calendar-based spraying. Most growers spray at least six times and eight times per year, in the dry season and wet season, respectively, irrespective of eggs, nymphs, or adults being present for both marcotted and initially pathogen-free trees. The reasons why many growers still prefer to use chemical pesticides include (1) poor knowledge about IPM due to limited education, (2) time required to assess the incidence of ACP and then implement appropriate IPM techniques, and (3) using pesticides is easier than IPM techniques (for example, using biological control) and they lack knowledge about the effect of chemical pesticides on natural enemies, the environment, and human health. 

### 7.7. Influence of Abiotic Factors

Monitoring environmental conditions and knowledge of such conditions are essential for anticipating the spread of the HLB and ACP within orchards and regions. Sarawak has a Köppen–Geiger tropical rainforest A*f* climate. Average annual rainfall in Kuching is 4165 mm; 2709 mm in the wet season from October to March, and 1456 mm in the dry season from April to September. Monthly averages during the wet season range from 339–701 mm, and in the dry season, from 206–277 mm. Leong et al. [108] attributed a decline in populations of eggs and adults on mandarin trees from October 1999 to January 2000 in their study near Kuching to frequent heavy rainfall dislodging eggs and nymphs from leaves. Low densities of eggs and adults were related to less frequent flush cycles and shorter flush durations during the dry season (<5 eggs and <10 adults) [108], but research is required to determine if the above ambient detrimentally high leaf temperatures after short diurnal rain showers or higher levels of UV light (particularly UV-B) during the dry season have detrimental effects on the psyllid (see Beattie, [46]). 

### 7.8. Biological Control of Asian Citrus Psyllid

Despite the application of insecticides in a field study conducted near Kuching, both primary parasitoids of ACP were able to parasitise nymphs and complete their development. Imidacloprid and MO appeared to have less impact on both parasitoids than foliar-applied carbamate, organophosphate, and pyrethroid insecticides [83]. Levels of parasitism recorded in the study ranged from 4.4 to 22.3%, and 2.6 to 35.3%, respectively, with three seasonal peaks annually on MO- and imidacloprid-treated trees, but the highest levels of parasitism occurred in the wet season [108]. Augmentative releases of mass-reared *T. radiata* and *D.* *aligarhensis* in citrus orchards should be encouraged by the SDOA for control of ACP nymphs, and carefully timed to coincide with the incidence of third- to fifth-instar nymphs in orchards.

Although populations of unidentified predators including coccinellids, lacewings, mantids, syrphid flies, and spiders were reported by Anon to increase in citrus orchards shortly after ACP was recorded in Sarawak in 1996 [110], scant information is known about their impact on the psyllid in sprayed and unsprayed orchards, or on unsprayed alternative hosts such as *M. paniculata.* The potential for using *O. smaragdina* in IPM programs for the psyllid and other citrus pests in orchards, particularly in small orchards (2–3 ha with 330 trees/ha), warrants investigation. Such use would require training of farmers to manage ant populations, including movement between trees, removing ants from orchards during harvesting, and use of wood ash to reduce the risk of ants biting workers in orchards. The impact of lacewings, including *Apertochrysa* spp. and *Chrysopa* spp. [111], on ACP warrants investigation for future research.

The most successful case of biological control of ACP using *T. radiata* and *D. aligarhensis* was reported in Réunion [110,112]. Recently, successful control of ACP has also been reported in major citrus-producing areas such as Brazil and California, USA [65,113,114]. Parasitoid mass production was initiated in the laboratory for mass releases in Brazilian conditions [115]. Periodic parasitoid releases were demonstrated by Diniz et al. [116]; the result was promising, significantly reducing the number of adult ACP in tests in commercials area in Brazil, and was included in the recommendations for HLB management. Based on the dispersal capacity and efficiency of the parasitoid, a release of 800–3200 parasitoids/ha, which results in a reduction of up to 80% of the total number of nymphs, was found to be most efficient in Brazil [117]. Parasitism by *T. radiata* was identified as a significant mortality factor, often exceeding 60% during periods of peak parasitoid activity [117].

### 7.9. Chemical Control of ACP and Spread of HLB

**Figure 3:**
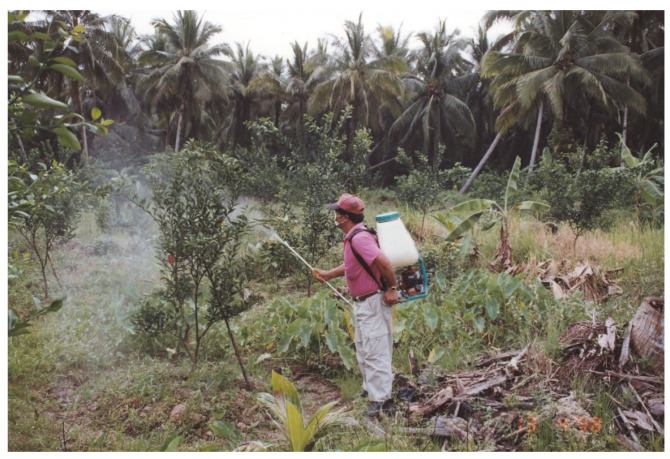
Spraying insecticide with a power knapsack sprayer in an HLB-free citrus orchard.

Multiple foliar insecticides are commonly used to suppress ACP populations in Malaysian orchards, but even six to fifteen sprays per year do not prevent the spread of the disease [20], as is the case elsewhere in Asia, where Catling [118] mentions application of four to thirty-five (mean 21.8) sprays, often ‘cocktails’ of two or more active ingredients, in twelve orchards surveyed in 1985, and seventy-three sprays in one orchard surveyed in 1991. Orchards based on marcotting may survive for 5–8 years if insecticides are applied thoroughly and frequently, and for <5 years if insecticide use is infrequent, poorly applied, or unsprayed, and >12 years in well-managed orchards, based on pathogen-free trees [86]. Contact insecticides typically do not control all life stages; the eggs and nymphs can hide inside new foliage, and the adults can fly. Furthermore, some insecticides show better efficacy against one stage over another. Insecticides currently registered for use include malathion, chlorpyrifos, trichlorfon, acephate, dimethoate, dicrotophos, methamidophos, monocrotophos, diazinon, triazophos, carbofuran, deltamethrin, cypermethrin, carbaryl, propoxur, pyriproxyfen, thiomethoxam, imidacloprid, acetamiprid/chlorpyrifos, cypermethrin/chlorpyrifos, and betacypermethrin/chlorpyrifos. The most commonly used are malathion, dimethoate, chlorpyrifos, trichlorfon, carbofuran, imidacloprid, deltamethrin, and cypermethrin/chlorpyrifos. Generally, organophosphate, carbamates, and pyrethroids require more frequent applications than neonicotinoids such as imidacloprid. Sprays are applied with knapsacks (hand-pump, battery-powered, and motorised) and stationary motorised units with long hoses on small farms, and drive-past and boat-mounted air-blast (with and without towers) and boom sprayers, in larger orchards (Figure 3).

Leong et al. [83] compared the impacts of four treatments over 3 years (January 1999 to December 2001) on the incidence of HLB in a honey tangerine orchard in Jemukan (1.5492° N, 110.6904° E), Samarahan Division, near Kuching: (i) unsprayed; (ii) 0.35% *v*/*v* aqueous emulsions of *n*C24 mineral oil (MO); (iii) triazophos (0.75 mL 40 EC/L of water) alternated with chlorpyrifos/cypermethrin (2.25 mL 500 EC/50 EC/L of water); and (iv) imidacloprid (0.5 mL 200 EC/L of water). The MO was applied weekly and the insecticides fortnightly. The sprays significantly reduced the incidence of ACP on new flush, and significantly reduced the incidence and spread of HLB in the orchard. Levels of HLB, as determined by PCR, rose over the 3 years to reach 42.2%, 9.4%, 11.4%, and 22.7% in the unsprayed, imidacloprid, MO, and triazophos/cypermethrin/chlorpyrifos treatments, respectively. High-quality MOs are suitable for use in organic farming, have limited negative effects on natural enemies [119,120], and pests and diseases do not become resistant to them [118]. Spray deposits reduce feeding and oviposition by ACP [85,121,122,123], and oviposition by dacine fruit flies (e.g., *Bacrocera dorsalis* (Hendel) and *B. tryoni* (Froggatt) (Diptera: Tephritidae)) [124,125,126], citrus leafminer (*Phyllocnistis citrella* Stainton (Lepidoptera: Gracillariidae) [127,128,129,130], and phytophagous mites (e.g., *Panonychus citri* (McGregor) (Acari: Tetranychidae) [131]. Wet and dry deposits of the oils can also kill ACP eggs and first-instar nymphs [93]. However, the cost of using MOs is higher than for insecticides and subject to fluctuations in world oil prices.

Foliar applications of broad-spectrum insecticides in Sarawak to mature (≥5 years old) trees in seasonally dry months from February–March and September–October, when flush growth is prolific and ACP populations rapidly increase, can reduce populations of the psyllid for relatively long intervals [83]. This indicates the importance of applying sprays at the end of rainy seasons as bud burst begins [83]. For example, single foliar applications of chlorpyrifos, triazophos, or cypermethrin to mature citrus trees in February–March significantly suppressed the ACP populations (both adults and nymphs) for up to a few months [83,85]. Nevertheless, HLB causes growers to panic and overuse pesticides in a desperate attempt to stop the threat. Growers may apply as many as six to fifteen foliar and one to two systemic treatments per year, from five chemical classes, in an effort to slow the speed of HLB in both marcotted and pathogen-free plantings [20].

Farmers under a subsidy scheme in Sarawak are entitled to receive free fertiliser and pesticides for 3 years after orchards are established. They are generally advised that trees ≤4 years old require at least one soil application of carbamate (e.g., carbofuran) per growing season, supplemented by three or four foliar applications of other insecticides, in order to achieve continuous protection from ACP. 

## 8. Conclusions

Asian citrus psyllid (ACP) is an important pest of citrus in Sarawak, elsewhere in Asia, and in the Americas, because it transmits the pathogen *C*Las, associated with the severe Asian form of huanglongbing (HLB), the most destructive and incurable disease of citrus. Concerted efforts must be made by growers, nurseries, and governments to minimise severe detrimental impacts on commercial citrus production and on the diversity of citrus germplasm. Governments must assist nurseries to supply certified HLB-free planting material, and help growers to diagnose the disease. 

Both biological and chemical control strategies are important components of an IPM program for ACP. These strategies, when combined with other practices (planting pathogen-free trees, removal of symptomatic trees, monitoring and control of the vector), greatly lowers the incidence and spread of HLB. Once the HLB incidence occurs and/or becomes widespread in an area, the chemical control alternative should be adopted as the main vector and disease management option, owing to advantages over the biological control option, if one does not consider the potential use of *O. smaragdina*. It is very important to protect citrus trees from HLB infection when they are young. The immediate use of preventive measures such as routine sampling, detection, monitoring, and removal of infected trees, and the judicious application of insecticides, such as chlorpyrifos, imidacloprid, and cyfluthrin/imidacloprid formulations, is required from budburst in each flush cycle, supplemented by the use of mineral oils, to reduce feeding by nymphs and adults (and thus acquisition of *C*Las), and oviposition by females. Augmentative releases of mass-reared *T. radiata* and *D.* *aligarhensis* in citrus orchards should be encouraged as alternatives to chemical insecticides. Unfortunately, the SDOA lacks the capacity to implement IPM in citrus due to shortage of staff, expertise, budget, and motivation. IPM-trained growers need further guidance in the form of citrus crop-specific modules for the monitoring of ACP populations, injury assessment in relation to growth stages, and advice on the proper means and timing of control. IPM farmer field schools can provide information about IPM and the use of pesticides that are environmentally acceptable. There is a pressing urgency to develop strategies that maintain effective use of currently available insecticides for sustainable ACP control. Ultimately, control of HLB in commercial citrus production will require the development of citrus cultivars that are tolerant or resistant to ACP and *C*Las. 

## Data Availability

No new data was generated for this paper. Data presented in the figures is available from the sources cited within the figure legends.
